# Pregnancy Outcomes in Women With Liver Cirrhosis: A National Prospective Cohort Study Using the UK Obstetric Surveillance System

**DOI:** 10.1111/1471-0528.18107

**Published:** 2025-03-13

**Authors:** Melanie Nana, Agata Majewska, Mussarat Rahim, Victoria Geenes, Caroline Ovadia, Marian Knight, Michael Heneghan, Catherine Williamson

**Affiliations:** ^1^ Department of Women and Children's Health King's College London London UK; ^2^ Department of Obstetrics and Gynaecology Institute of Mother and Child Warsaw Poland; ^3^ Department of Hepatology King‘s College Hospital London UK; ^4^ National Perinatal Epidemiology Unit, Nuffield Department of Population Health University of Oxford Oxford UK; ^5^ Institute of Reproductive and Developmental Biology Imperial College London London UK

**Keywords:** ALBI score, cirrhosis, liver disease, morbidity, mortality, neonatal, perinatal

## Abstract

**Objective:**

Describe maternal/fetal outcomes of pregnant women with cirrhosis.

**Design:**

Prospective, national cohort study utilising the UK Obstetric Surveillance System between 1st June 2017 and 30th November 2020.

**Setting:**

UK.

**Population:**

Pregnant women with cirrhosis.

**Methods:**

Rates of adverse perinatal outcomes were compared with published rates for uncomplicated pregnancies. The prediction of adverse pregnancy outcomes by albumin‐bilirubin (ALBI) score was determined.

**Main Outcome Measures:**

Maternal and fetal outcomes.

**Results:**

52 eligible cases were reported (denominators represent available data for each outcome). Commonest causes included autoimmune hepatitis (12/50 (24.0%)), cholestatic disease (9/50 (18.0%)) and viral disorders (8/50 (18.0%)). Maternal decompensation occurred in seven women. Worst ALBI score predicted decompensation and maternal ICU admission (AUROC 0.80 (*p* = 0.03) and 0.81 (*p* = 0.03), respectively). Untreated varices were associated with increased rates of variceal bleed (*p* = 0.01). No women died. There were 42 live births (51.2% preterm), one stillbirth, and two neonatal deaths. The worst ALBI score in pregnancy predicted pre‐term birth (AUROC 0.74 (*p* = 0.03)). Compared to a healthy population, women with cirrhosis were at increased risk of cholestasis in pregnancy (OR 29.4, 95% CI 13.8–61.6, *p* < 0.001), ICU admission (OR 42.5,95% CI 15.2–118.8, *p* < 0.001), pre‐term birth (OR 13.2, 95% CI 7.1–24.4, *p* < 0.001), and babies with low birth weight (OR 12.0, 95% CI 6.5–22.0, *p* < 0.001), neonatal intensive care unit admission (OR 4.4, 95% CI 2.4–8.2, *p* < 0.001) and perinatal mortality (OR 15.8, 95% CI 4.9–51.3, p < 0.001).

**Conclusion:**

Women with cirrhosis and their babies are at increased risk during pregnancy. The ALBI score predicts maternal decompensation, ICU admission, and pre‐term birth.

## 1 Introduction

Cirrhosis is estimated to affect 45/100,000 women of childbearing age [1]. The most common causes of cirrhosis in women globally include hepatitis B and C viruses, non‐alcoholic steatohepatitis, and alcohol‐associated liver disease. Autoimmune and genetic disorders also contribute to the aetiology of cirrhosis. Pregnancy has previously been considered an uncommon occurrence in women with liver cirrhosis owing to decreased natural fertility rates [2, 3] and a paucity of data exists on pregnancy outcomes and optimal management in this group. However, with improved screening and treatment of cirrhosis, and increased availability of assisted reproductive techniques, more women with the condition in pregnancy are being encountered [4, 5, 6, 7, 8, 9]. Lack of data may limit the ability of clinicians to appropriately counsel women with cirrhosis regarding pregnancy and in determining the best evidence‐based management.

Historical studies have reported higher rates of maternal and neonatal mortality for women with cirrhosis and their babies [1, 10, 11], with those with portal hypertension and oesophageal varices considered to be at the highest risk [9, 12]. More recent studies suggest that maternal mortality is falling [7, 13, 14], although none have been large enough to accurately quantify the risks. In a recent systematic review and meta‐analysis [9], including 2912 pregnancies in women with cirrhosis, the maternal mortality rate was reported as 0.89% and was most commonly associated with variceal haemorrhage. Beyond mortality, other documented maternal complications for women with cirrhosis include higher rates of anaemia, induction of labour, caesarean section, post‐partum haemorrhage, increased puerperal infections, cholestasis, pre‐eclampsia, and placental abruption [8, 9, 11, 13]. Fetal complications are reported to include miscarriage, small‐for‐gestational age, pre‐term delivery, neonatal distress, and intrauterine growth restriction [8, 9, 10, 11, 13]. Live birth rates are poorly described, with rates between 58% and 100% reported [15, 16, 17]. Rates of neonatal death are reported to be between 0% and 8.3% [8, 10, 11, 14, 16, 18, 19]. Previous studies have demonstrated that prediction models calculated in the pre‐conception period can assist in the prediction of maternal decompensation in women with pre‐existing cirrhosis in pregnancy [7], but their utility has not been assessed during pregnancy.

There are currently no prospective studies in cirrhosis and pregnancy. We therefore aimed to determine the UK incidence of cirrhosis in pregnancy and to further describe maternal and fetal outcomes in this group using the UK Obstetric Surveillance System (UKOSS). In addition, we aim to determine the utility of the albumin‐bilirubin (ALBI) score when calculated during pregnancy to predict outcomes.

## 2 Methods

We performed a national, prospective, observational cohort study between 1st June 2017 and 30th November 2020 using UKOSS to study liver cirrhosis in pregnancy. UKOSS is a national research platform used to study rare disorders of pregnancy that utilises a monthly case collection scheme and includes all 193 consultant‐led obstetric units in the UK. Data are collected during hospital admission; thus, patients are not contacted directly, and no personally identifiable information is collected. It is anticipated that all women with cirrhosis in pregnancy in the UK receive consultant‐led care, and thus it is expected that this study has identified all cases within the UK birth cohort during the data collection period. The study was approved by the London Multi‐centre Research Ethics Committee (04/MRE02/45).

### 2.1 Case Definition

Cases were defined as pregnant women with an established history of cirrhosis defined by either confirmation on liver biopsy or on the basis of radiological findings (nodular liver with enlarged spleen on ultrasound or axial imaging or both) with either a history of complications of liver disease (ascites, variceal bleeding, encephalopathy, pervious bacterial peritonitis) or supportive laboratory findings (low platelets, low albumin, prolonged prothrombin time), or International Normalised Ratio (INR).

### 2.2 Data Collection

Women who met the criteria were identified by the obstetrician or physician responsible for their care. Reporting clinicians were then asked to complete a data collection form. Anonymised data were collected on women's demographic details, previous obstetric history, medical history, current pregnancy, delivery, and maternal and infant outcomes. Large‐for‐gestational age and small‐for‐gestational age outcomes were determined using the intergrowth calculator [20]. Blood test results, including the ‘worst’ and pre‐delivery albumin, bilirubin, creatinine, platelets, haemoglobin, prothrombin time, and alanine transaminase, were documented by the clinician filling in the data collection form. Due to the nature of the study, no follow‐up data are available post‐delivery.

### 2.3 Statistical Analysis

Statistical analysis was performed using Stata 17.0 (Stata Corporation, TX, USA) and GraphPad Prism v9 (GraphPad software, CA, USA). Continuous variables were presented as medians and interquartile ranges, categorical variables as frequencies and percentages. For the analysis of continuous variables, Mann–Whitney *U*‐tests were used, and for categorical variables, Fisher exact test was applied. When the numerator was 0, we used exact logistic regression to calculate an odds ratio. Binomial distribution was used to calculate the confidence interval around the main measure of cirrhosis incidence.

Prediction of adverse pregnancy outcomes by the ALBI score was determined by calculating the area under the receiver operating characteristic curve (AUROC). Maternal decompensation was defined as the development of new jaundice, ascites, or hepatic encephalopathy. Rates of adverse perinatal outcomes for women with cirrhosis and their neonates were compared with published rates of adverse perinatal outcomes in uncomplicated pregnancies or at population levels (we aimed for UK published rates in the same time period (2017–2020)), if these were not available we prioritised UK data outside of this time frame and in cases where UK data were not available we searched for any available published rates internationally), and compared using the chi‐square test with Yates' correction and woolflogit to calculate the 95% confidence interval (CI) (graphpad prism). A *p*‐value was considered significant if less than 0.05.

## 3 Results

### 3.1 Incidence and Maternal Characteristics

During the 3‐year and 6‐month study period, there were 58 unique cases of cirrhosis reported to UKOSS; all notified cases met entry criteria, three were excluded as they had received liver transplantation prior to pregnancy and were therefore mistakenly reported, in two cases it was unclear as to whether they had received liver transplantation, and one case was excluded as essential data were not reported (Figure S1). 52 cases were therefore included in the study in an estimated 2,256,366 maternities, giving an estimated incidence of 2.3 per 100,000 maternities (95% CI 0.15–0.35 per 10 000). Maternities were defined as a pregnancy resulting in the birth of one or more babies (including stillbirths). The demographics and disease characteristics of the women are described in Table 1. The denominator represents the total number of patients in which each outcome was available.

**TABLE 1:** Demographics and disease characteristics of women with cirrhosis in pregnancy.

Maternal age at first appointment (years), median (IQR) (*n* = 49)	34 (29–37)
Pre‐pregnancy BMI, median (IQR) (*n* = 49)	25.44 (22.3–28.3)
Ethnicity (*n* = 50)	
White, *n* (%)	37 (74.0%)
Asian, *n* (%)	6 (12.0%)
Black, *n* (%)	5 (10.0%)
Mixed, *n* (%)	1 (2.0%)
Other, *n* (%)	1 (2.0%)
Cirrhosis confirmed on	
Liver biopsy, *n* (%)	19/38 (50.0%)
CT/MRI/US, *n* (%)	32/39 (82.1%)
Presence of portal hypertension (*n* = 46), *n* (%)	27 (58.7%)
Diagnosed prior to pregnancy, *n* (%)	9/14 (64.3%)
Diagnosed in pregnancy, *n* (%)	5/14 (35.7%)
Oesophageal varices diagnosed before pregnancy (*n* = 46), *n* (%)	19 (41.3%)
Grade I, *n* (%)	9 (47.4%)
Grade II, *n* (%)	2 (10.5%)
Grade III, *n* (%)	0 (0.0%)
Not known, *n* (%)	8 (42.1%)
Treatment of variceal bleed prior to pregnancy (*n* = 19), *n* (%)	7 (36.8%)
Band ligation, *n* (%)	5/6 (83.3%)
Injection sclerotherapy, *n* (%)	1/6 (16.7%)
History of gallstones (*n* = 45), *n* (%)	6 (13.3%)

*Note:* The denominator represents the total number of patients in which each outcome was available.

Abbreviations: BMI, body mass index; CT, computer tomography; IQR, interquartile range; MRI, magnetic resonance imaging; US, ultrasound.

The cause of cirrhosis was documented in 50 women; the most common causes included autoimmune hepatitis 12/50 (24.0%), cholestatic liver disease 9/50 (18.0%) and viral disorders 8/50 (18.0%) (Figure S2). Median duration since cirrhosis diagnosis was 3.44 years (IQR 0.7–7.0). A total of 27/46 (58.7%) had a confirmed diagnosis of portal hypertension; where the information was available, this diagnosis was made prior to pregnancy in 64.3% of patients and during pregnancy in 35.7%. In total, 19/46 (41.3%) had confirmed oesophageal varices, of which 7/19 (36.8%) had been treated.

Of the 52 women for whom obstetric history was reported, 28/52 (53.9%) were multiparous; of these, four women had experienced hepatic deterioration in a previous pregnancy, one woman had a stillbirth, four had a preterm birth, and five women had babies that required admission to a neonatal intensive care unit.

### 3.2 Pregnancy Outcomes

Only 17/42 (40.5%) of women received pre‐pregnancy counselling. The majority of women, 49/52 (94.2%) had spontaneous conception, and all were singleton pregnancies. The medications taken during pregnancy are described in Table 2; most commonly prescribed were corticosteroids (*n* = 9), immunosuppressive therapy (*n* = 9) and beta‐blockers as primary prophylaxis for variceal bleed (*n* = 9). Maternal blood test results are illustrated in Figure S3, with worst and pre‐delivery values plotted.

**TABLE 2:** Pregnancy outcomes of women with cirrhosis in pregnancy.

Type of conception (*n* = 52)	
Spontaneous, *n* (%)	49 (94.2%)
Assisted, *n* (%)	3 (5.8%)
Miscarriage (*n* = 51), *n* (%)	3 (5.9%)
Multifetal pregnancy, *n*	0
Pre‐exiting treatment continued during pregnancy	
UDCA (*n* = 46), *n* (%)	9 (19.6%)
Started prior to pregnancy	5
Started in pregnancy	4
Steroids (*n* = 49), *n* (%)	9 (18.4%)
Started prior pregnancy	9
Started in pregnancy	0
Vitamin K (*n* = 46), *n* (%)	6 (13.0%)
Immunosuppression (*n* = 46), *n* (%)	9 (19.6%)
Azathioprine, *n* (%)	5 (55.6%)
Tacrolimus, *n* (%)	2 (22.2%)
Beta blockers (*n* = 51), *n* (%)	9 (17.7%)
Spironolactone (*n* = 51), *n* (%)	3 (5.9%)
Proton‐pump inhibitors (*n* = 51), *n* (%)	4 (7.8%)
Pregabalin (*n* = 51), *n* (%)	1 (2.0%)
Deterioration in current pregnancy (*n* = 41), *n* (%)	6 (14.6%)
Symptoms/signs during pregnancy (*n* = 52):	
Pruritus, *n* (%)	12 (23.1%)
Jaundice, *n* (%)	2 (3.8%)
Ascites, *n* (%)	3 (5.8%)
GI bleeding, *n* (%)	2 (3.8%)
Endoscopy during pregnancy (*n* = 49), *n* (%)	26 (53.1%)
Varices, *n* (%):	15 (57.7%)
Grade I‐II, *n* (%)	12/14 (85.7%)
Grade III, *n* (%)	2/14 (14.3%)
Variceal treatment in pregnancy, *n* (%)	9 (60.0%)
ICU admission (*n* = 46), *n* (%)	4 (8.7%)
Duration of ITU admission (days), median (IQR)	2 (1.5–3.5)
Maternal death, *n*	0
Encephalopathy during pregnancy (*n* = 50), *n* (%)	1 (2.0%)
Variceal bleeding during pregnancy (*n* = 51), *n* (%):	2 (3.9%)
Banding, terlipressin, *n* (%)	1 (50.0%)
Blood transfusion, *n* (%)	1 (50.0%)
Liver transplant during pregnancy, *n*	0
Pregnancy complications (*n* = 52)	
PIH, *n* (%)	1 (1.9%)
PE, *n* (%)	1 (1.9%)
Cholestasis, *n* (%)	9 (17.3%)
GDM, *n* (%)	3 (5.8%)

*Note:* The denominator represents the total number of patients in whom each outcome was available.

Abbreviations: GDM, gestational diabetes mellitus; GI, gastrointestinal; ITU, intensive care unit; PE, preeclampsia; PIH, pregnancy induced hypertension; UDCA, ursodeoxycholic acid.

The maternal outcomes are described in Table 2. Maternal decompensation occurred in six women in total. This included the development of new jaundice in two women; one with cirrhosis secondary to congenital/genetic disease and the other secondary to viral disease. The cause of maternal decompensation was new ascites in three women, of whom two had cirrhosis secondary to viral disorders and one had autoimmune hepatitis. The cause of maternal decompensation was the development of new encephalopathy in the final patient, who had a history of nodular regenerative hyperplasia. Variceal bleeding occurred only in women who had not previously received endoscopic treatment for varices; one had alcohol‐related liver disease and the second had vascular liver disease.

Untreated, compared to treated, varices were associated with increased rates of intensive care unit admission (ITU) (3/6 vs. 0/9, OR ∞ (95% CI 1.6 to ∞), *p* = 0.044), but were not associated with increased risk of maternal deterioration (1/6 vs. 3/9, OR 0.4 (0.0 to 4.1) *p* = 0.604) or variceal bleed (2/6 vs. 0/9, OR ∞ (0.9 to ∞), *p* = 0.143). Similarly, patients who had required treatment for varices pre‐pregnancy had an association with increased risk of variceal bleed in pregnancy (2/6 vs. 0/41, OR ∞ (4.3 to ∞), *p* = 0.014) but had no significantly increased risk of ITU admission (2/7 vs. 2/30, OR 6.8 (1.0 to 49.3), *p* = 0.118) or maternal deterioration (1/5 vs. 5/41, OR 1.8 (0.0 to 17.4), *p* = 0.520).

In contrast, compared to 37 women with no prior portal hypertension, a history of portal hypertension diagnosed prior to pregnancy (*n* = 9) was not associated with ITU admission, maternal deterioration, variceal bleed, or postpartum haemorrhage (2.2 (0.0 to 19.5), *p* = 0.488, 1.8 (0.3 to 10.6), *p* = 0.613, 4.4 (0.0 to ∞), *p* = 0.364 and 1.0 (0.0 to 5.4), *p* > 1.000, respectively). Deterioration in a previous pregnancy was not associated with deterioration in the current pregnancy (2/4 vs. 4/29, OR6.3 (0.8 to 48.3), *p* = 0.142).

The most commonly reported symptom in pregnancy was pruritus (*n* = 12). Of these 12 women, seven received a diagnosis of cholestasis in pregnancy. An additional two patients who did not present with itch were diagnosed with cholestasis of pregnancy. Out of these nine patients, four had underlying cholestatic disease: one vascular, one viral, and three congenital liver diseases. Other gestational complications are outlined in Table 2. During the pregnancy, 26/49 (53.1%) women had an endoscopy; 9/26 (34.6%) of these underwent uncomplicated banding. Four women required intensive care unit admission. No women required liver transplantation, and there were no maternal deaths.

### 3.3 Obstetric Outcomes

Obstetric outcomes are described in Table 3. There were 42 live births and one stillbirth (outcomes were not available for nine pregnancies); outcome data were missing for the remaining. Twenty‐one of 41 (51.22%) were preterm (in one baby the gestation at birth was not reported). It was not possible to determine from the data what proportion of preterm births were spontaneous or iatrogenic. Median gestation at birth was 36 + 4 weeks' gestation (IQR 34 + 6–38 + 1 weeks). In total, 20/41 (48.78%) of babies were born by caesarean section. 12/41 (29.27%) women had a postpartum haemorrhage.

**TABLE 3:** Obstetric and neonatal outcomes.

Obstetric outcomes	
All births, *n*	43
Gestation at birth (weeks + days), median (IQR)	36 + 4 (34 + 6–38 + 1)
Preterm birth (*n* = 41), *n* (%)	21 (51.2%)
Mode of birth (*n* = 41)	
Caesarean section, *n* (%)	20 (48.8%)
Spontaneous vaginal birth, *n* (%)	18 (43.9%)
Assisted vaginal birth, *n* (%)	4 (9.8%)
Est. Blood Loss, median (IQR) (*n* = 11)	1000 (700–1700)
Postpartum haemorrhage (*n* = 41), *n* (%)	12 (29.3%)
Placental abruption (*n* = 52), *n* (%)	1 (1.9%)
Neonatal outcomes	
Birthweight (g) *n* = 42, median (IQR)	2615 (2300–3190)
LBW, *n* (%)	19/42 (45.2%)
SGA, *n* (%)	7/42 (16.7%)
LGA, *n* (%)	5/42 (11.9%)
Stillbirth, *n* (%)	1/42 (2.4%)
MSAF, *n* (%)	4/42 (9.5%)
NICU admission, *n* (%)	18/42 (42.9%)
Prematurity	5/14 (35.7%)
Hypoglycemia	2/14 (14.3%)
Sepsis	1/14 (7.1%)
Isoimmunisation of the newborn	2/14 (14.3%)
Consequences of maternal substance abuse	2/14 (14.3%)
Low APGAR score	1/14 (7.1%)
Neonatal death, *n* (%)	2/41 (4.9%)
Congenital abnormality (hypothyroidism), *n* (%)	1/40 (2.5%)

*Note:* The denominator represents the total number of patients in whom each outcome was available. For preterm birth, the denominator is 41, as one baby was stillborn and for one, the gestation at the week of delivery was not reported.

Abbreviations: IQR, interquartile range; LBW, low‐birth‐weight; LGA, large‐for‐gestational‐age; MSAF, meconium stained amniotic fluid; NICU, neonatal intensive care unit; SGA, small‐for‐gestational‐age.

Neonatal outcomes are also described in Table 3. Median birthweight was 2615 g (IQR 2300–3190). Nineteen out of 43 (44.2%) of the neonates had low birth weight; 7/42 (16.7%) were small for gestational age (using the intergrowth calculator [20]); 5/42 (11.9%) were large for gestational age; 1/42 (2.4%) were stillborn, and 2/41 (4.9%) neonates died; both deaths were considered to be a consequences of prematurity. One neonate had congenital hypothyroidism.

The ALBI score was available for 36 patients. The median weeks of gestation when the worst ALBI score occurred was 22 weeks and 6 days (IQR 13 weeks and 1 day to 33 weeks and 4 days). The median worst ALBI score was −1.80 (minimum −2.81 and maximum −0.13). The worst ALBI score in pregnancy predicted maternal decompensation, AUROC 0.80 (*p* = 0.03) (Figure S4) and predicted maternal ICU admission, AUROC 0.82 (*p* = 0.03) (Figure S4). The worst ALBI score in the pregnancy predicted pre‐term birth AUROC 0.74 (*p* = 0.03), Figure S4. Worst ALBI did not predict the risk of fetal growth restriction (*p* = 0.91), small‐for‐gestational‐age (*p* = 0.72), low‐birth‐weight (*p* = 0.10) or neonatal intensive care unit admission (*p* = 0.27).

### 3.4 Comparison to Healthy Reference Populations

When compared to UK healthy reference populations (Table 4), there was a relationship between pregnant women with cirrhosis and increased risk of preterm birth (*p* < 0.0001), caesarean section (*p* = 0.005), and babies with low birth weight (*p* < 0.0001), neonatal intensive care admission (*p* < 0.0001), stillbirth (*p* = 0.024), neonatal death (*p* = 0.002) and perinatal mortality (*p* = 0.001). There was also a relationship between women with cirrhosis and being more likely to develop cholestasis in pregnancy (*p* < 0.0001) and to be admitted to an intensive care unit (*p* < 0.0001).

**TABLE 4:** Comparison of outcomes compared to the healthy background population.

Adverse perinatal outcome	Reference population description	Rate in reference population, % (affected/total)	UKOSS Study cohort description	Rate in UKOSS Study (affected/total)	Significance *p*
Preterm birth	Births in England and Wales 2020 [21]	7.4% (45 116/613 231)	41 infants born at least 24/40 weeks of gestation	51.2% (21/41)	*p* < 0.0001
Caesarean section	National Maternity Statistics, England 2016–17 [22]	27.8% (83 972/636401)	41 infants born at least 24/40 weeks of gestation	48.8% (20/41)	*p* = 0.005
LBW	Births in England and Wales 2020 [21]	6.5% (39 672/613 936)	42 infants	45.2% (19/42)	*p* < 0.0001
LGA (Intergrowth‐21st Growth Chart)	15 European countries LGA incidence in patients ≥ 33 weeks of gestation (2016–2017) [23]	5.4% (79 251/1 477 840)	42 infants born at least 33/40 weeks of gestation	11.9% (5/42)	*p* = 0.073
NICU admission	BLISS 2016 [22]	14.5% (100 762/696 271)	42 infants born at least 24/40 weeks of gestation	42.9% (18/42)	*p* < 0.0001
Stillbirth	UK MBRRACE 2019 [24]	0.3% (2399/716 825)	42 total births reached at least 24/40 weeks of gestation	2.4% (1/42)	*p* = 0.024
NND	UK MBBRACE data from 2019 [24]	0.2% (1158/716 825)	41 live births	4.9% (2/41)	*p* = 0.002
Perinatal mortality	UK MBRRACE 2019 [24]	0.5% (3557/716 825)	42 total births	7.3% (3/41)	*p* = 0.001
PE	CALIBRE 2019 [25]	2.4% (31 478/1 303 365)	52 maternities	1.9% (1/52)	*p* = 1.000
GDM	IDF Diabetes Atlas 2021, UK [26]	20.6%	52 maternities	5.8% (3/52)	*p* = 0.005
Cholestasis	Birmingham UK 1995–1997 [27]	0.7% (73/10335)	52 maternities	17.3% (9/52)	*p* < 0.0001
ICU admission	National Maternity and Perinatal Audit (2015–2016) [28]	0.2% (1529/683955)	46 maternities	8.7% (4/46)	*p* < 0.0001

*Note:* Compared to background population rates using binominal probability test.

Abbreviations: CI, confidence interval; GDM, gestational diabetes mellitus; ICU, intensive care unit; LBW, low‐birth‐weight; LGA, large for gestational age; MSAF, meconium‐stained amniotic fluid; NICU, neonatal intensive care unit; NND, neonatal death; PE, pre‐eclampsia.

## 4 Discussion

### 4.1 Main Findings

This 3.5‐year UKOSS study provides prospective data on 52 women with cirrhosis in pregnancy and is the first to prospectively define the UK incidence of cirrhosis in pregnancy. Key findings included an increased risk of admission to an intensive care unit in those with untreated varices and an increased incidence of intrahepatic cholestasis in our population. When compared to the background pregnant population, we report increased rates of maternal intensive care unit admission, rates of preterm birth, low birth weight, neonatal intensive care admission, and perinatal mortality consistent with the previously published literature. This study has shown that the calculation of the ALBI score during pregnancy can predict adverse maternal and perinatal outcomes.

### 4.2 Interpretation

We report an incidence of 2.3 per 100,000 maternities, which is less than previously reported in the literature [17, 29]. The most common underlying causes in this cohort included autoimmune hepatitis, cholestatic liver disease, and viral disorders. These causes differ from a recent North American study, which described non‐alcoholic fatty liver disease (now called metabolic dysfunction‐associated steatotic liver disease (MASLD)) as the emerging leading cause of cirrhosis in their pregnant cohort (reflecting the underlying aetiology in 71% of cases); we have not demonstrated such trends in this UK study [21]. A possible explanation for the lower rate of cirrhosis reported in this study may be that women with MASLD‐associated cirrhosis were not all ascertained, that there are lower rates of diagnosis in the UK, or the UK population of childbearing age may have lower rates of MASLD than north America [30]. We recognise that the estimated prevalence of maternal obesity in Europe is lower than that of North America (prevalence in Europe is 12.1% [95% CI 11.2–12.9%] compared to 18.7% [95% CI 15.0–23.2%] in North America) and therefore predict that with increasing UK obesity rates we may in time follow the American trend [23].

The median age of women in the study was 34 years old, which is 3.3 years above the average age of mothers at childbirth in England and Wales [22]. This perhaps reflects a longer period to conception for women with cirrhosis. Early studies in this field have suggested lower fertility rates in women with cirrhosis; however, in our study, 94% of women conceived spontaneously (accepting the caveat that we would not capture women who had tried to conceive but failed and did have assisted conception). Consistent with this, fertility rates were not reduced in women with cirrhosis of all aetiologies apart from alcohol‐related cirrhosis in a recent Canadian study that used national health care records [13]. This has important implications in terms of pre‐pregnancy counselling (PPC) which was only received by one‐third of the patients in this study. PPC has been demonstrated to improve outcomes in women with other chronic medical conditions [24] and offers the opportunity to optimise the underlying medical condition, discuss appropriate medications, and counsel women regarding relevant risks prior to planned pregnancy. Thus, PPC should be offered to all women with cirrhosis; this is a recommendation in the EASL Clinical Practice Guideline on the management of liver diseases in pregnancy [25] and will be recommended in similar international guidance, for example, the upcoming International Federation of Gynaecology and Obstetrics guideline. The medications prescribed to women in our cohort are largely compatible with pregnancy. Although this was not the case in this study, it should be recognised that some women with cirrhosis may be taking medications considered contraindicated in pregnancy, including mycophenolate mofetil [26], and this should be discussed in advance of pregnancy.

In this study, previous deterioration in pregnancy was not associated with deterioration in current pregnancy, accepting the low statistical power of our study to detect such an association. Potential theories as to why this was the case include women being considered high risk in a future pregnancy, thus having more intensive monitoring, and a previous adverse event meaning that it is likely these women will be prioritised for PPC. This is supported by the fact that 4/5 women who had a deterioration in a previous pregnancy had received PPC prior to a future pregnancy. This supports a focus on preparation for pregnancy, including consideration of present disease optimisation and not relying on previous pregnancy outcomes to predict disease course in this pregnancy. Previous studies have demonstrated that the model for end‐stage liver disease (MELD) score, and the related UKELD score, can assist in predicting the likelihood of maternal decompensation in women with pre‐existing cirrhosis in pregnancy [16]. Furthermore, the MELD, UKELD, albumin‐bilirubin (ALBI) scores, and the pre‐conception aspartate aminotransferase (AST)‐to‐platelet ratio index (APRI) are of value to predict adverse pregnancy outcomes, including preterm birth and perinatal mortality [7]. The EASL Clinical Practice guideline recommends the use of risk scores to characterise the risk profile of pregnant women with cirrhosis prior to conception [25]. This study has shown that the calculation of the ALBI score when a woman is pregnant is also of value to predict adverse maternal and perinatal outcomes. This is of particular relevance considering the number of unplanned pregnancies in the UK, and a score that can be used in pregnancy provides an opportunity to risk stratify even if the opportunity in the pre‐conception period is missed. The authors recommend that this be incorporated into future European and international guidance.

In total, 59% and 41% of women had a history of portal hypertension and known oesophageal varices, respectively, prior to pregnancy, both of which have been reported as being associated with poor outcomes [9, 12]. Complications of portal hypertension resulting from variceal bleeding and hepatic failure have been reported to be as high as 30%–50% in pregnancy [12] and in the recent meta‐analysis, the most common cause of maternal mortality was variceal haemorrhage [9]. In our study, we did not see increased rates of decompensation in those with portal hypertension or untreated varices, but a clear increased risk of bleeding was seen in those with untreated varices. In total, we reported 26 cases where women had endoscopy during the pregnancy, of whom almost half had uncomplicated banding. In our cohort, variceal bleeding was reported in two cases, neither of whom had previous variceal treatment. This therefore supports EASL guidance to perform screening endoscopy within a year prior to conception, or in cases where this has not occurred, undertake a screening endoscopy in the second trimester of pregnancy to assess for clinically significant varices so that appropriate primary prophylaxis and endoscopic management can be provided if required [25].

In terms of other maternal outcomes, an association was made with an increased risk of developing cholestasis in pregnancy, in keeping with other studies [27]. Women should therefore be advised that if they develop pruritus during pregnancy, they should be investigated and managed appropriately for cholestasis [28]. We did not identify a relationship between cirrhosis and increased rates of pre‐eclampsia or gestational diabetes mellitus above the background population rates. Increased rates of pre‐eclampsia have previously been reported in the literature; [9, 13] the variation in risk for this metabolic complication may relate to the varying underlying aetiology of the cirrhosis in different cohorts. For example, in the North American study, which reported the underlying aetiology to be non‐alcoholic fatty liver disease in 71% of cases, a composite of gestational hypertension, eclampsia, pre‐eclampsia, and hypertension, elevated liver enzymes, and low platelets described as ‘hypertensive complications’ was demonstrated to be higher in the patients with compensated cirrhosis compared to a healthy background population (11.2% vs. 8.8%, *p* < 0.001) [13]. It is also possible that women with cirrhosis were identified as being at increased risk of pre‐eclampsia and prophylactic low‐dose aspirin treatment was given, as this has been shown to reduce the likelihood of preterm pre‐eclampsia [31].

A history of cirrhosis in our cohort was associated with an increased risk of being admitted to an intensive care unit compared to the background population. For women identified to be at higher risk of decompensation, this should be discussed prior to pregnancy, and for pregnant women, the clinical teams can make preparations to minimise disruption for mother and baby following separation post‐delivery. No women required liver transplantation, and there were no maternal deaths. In a recent systematic review and meta‐analysis [9], that included 2912 pregnancies in women with cirrhosis from 1982 to 2000, the overall mortality rate was 0.89%, with a demonstration of a decreasing trend over time. Our results are therefore in keeping with such improvements and can further inform counselling of these women.

Just over half of the babies were born by caesarean section, a higher rate of operative delivery than in the background population, but in keeping with other literature [9, 19, 32, 33]. Rates of preterm birth [9, 11], low‐birth weight [11, 19], neonatal intensive care admission, and perinatal mortality [11] were also above the background population rates, also consistent with previous studies. The rate of congenital abnormality (2.5%) is comparable with the background incidence of 3% [34].

### 4.3 Strengths and Limitations

A strength of this study is that the data were collected prospectively from all UK obstetrician‐led units. The study was advertised through the established UKOSS system and additionally highlighted through emailing UK hepatology networks. The prospective nature of this study enabled us to collect data that has not previously frequently been collected in the literature, such as rates of spontaneous conception, which will inform counselling of these women in the future.

The study is limited by small numbers, which limit statistical analysis. We had no robust method to cross‐check case ascertainment, and it is possible some cases were not identified or missed. As UKOSS is an anonymous reporting system, it is not possible to go back to the reporters to collect missing information. In addition, reference UK population ranges were not available for all outcomes; we therefore had to use non‐UK data in some cases. It is difficult to ascertain disease control in these cases, which is likely to influence maternal and fetal outcomes and should be a focus of future work. Finally, we did not have the relevant biochemistry data to enable us to calculate the other prediction scores, and we recommend this be a focus of future work.

## 5 Conclusion

This is the first prospective, national cohort study of cirrhosis in pregnancy. In the UK population, the underlying aetiology of cirrhosis in pregnant women differs from a recent north American study, with fewer cases of MASLD. Cirrhosis and pregnancy are associated with increased rates of adverse maternal and fetal outcomes, including maternal ICU admission, increased perinatal mortality and preterm birth. Furthermore, women with untreated varices had increased rates of variceal haemorrhage, underlying the importance of pre‐pregnancy counselling to enable interventions to improve outcomes.

## Author Contributions

C.W. conceived the study. V.L.G., C.O., M.K., M.H., and C.W. designed the UKOSS data collection sheet. M.R., M.H., and C.W. publicised and contributed to data collection strategies. M.N., A.M., and C.O. performed statistical analysis. M.H. and C.W. reviewed data analysis. All authors contributed to manuscript writing and review.

## Ethics Statement

The study was approved by the London Multi‐centre Research Ethics Committee (04/MRE02/45).

## Conflicts of Interest

The authors declare no conflicts of interest.

## Supporting information


Figure S1.

Figure S2.

Figure S3.

Figure S4.

## Data Availability

The data that support the findings of this study are available from the corresponding author upon reasonable request.
