# Supplementary material for: Pregnancy Outcomes in Women With Liver Cirrhosis: A National Prospective Cohort Study Using the UK Obstetric Surveillance System
Source: BJOG. 2025 Mar 13;132(7):935–43. doi: 10.1111/1471-0528.18107 (PMC12051225; doi:10.1111/1471-0528.18107)
Supplement: Supplementary file 1 — Figure S1. [file BJO-132-935-s004.docx]

Total pregnancies in study period
*N = 2 280 000*

Excluded, *N = 1:*- Collection form not received, *N = 1*

Pregnancies that meet inclusion criteria

*N = 52*

Data collection forms received

*N = 57*

Cases notified
*N = 58*

Excluded, *N = 6:
-* Received liver transplantation prior pregnancy, *N = 3
-* Unclear whether received liver transplantation prior pregnancy, *N = 2*

- Autoimmune hepatitis, *N = 13*

- Cholestatic liver disease, *N = 9*

- Viral disorders, *N = 8*

- Congenital/Genetic including MDR3 deficiency, *N = 8*

- Alcohol-related liver disease,
*N = 6*

- Vascular liver disease, *N = 4*

- Drug induced liver disease,
*N = 2*

- NASH, *N = 1*

- Other liver diseases, *N = 2*

Supplementary Figure 1: Flow diagram showing the case ascertainment and completeness of reporting.
