# Supplementary material for: Pregnancy Outcomes in Women With Liver Cirrhosis: A National Prospective Cohort Study Using the UK Obstetric Surveillance System
Source: BJOG. 2025 Mar 13;132(7):935–43. doi: 10.1111/1471-0528.18107 (PMC12051225; doi:10.1111/1471-0528.18107)
Supplement: Supplementary file 2 — Figure S2. [file BJO-132-935-s003.docx]

Supplementary Figure 2: Underlying causes of cirrhosis. Abbreviations: MASLD – metabolic dysfunction-associated steatotic liver disease; MDR3 – multidrug resistance protein 3. Other liver diseases included nodular regenerative hyperplasia (n=1), schistosomiasis (n=1) and cryptogenic liver disease (n=1).
