# Supplementary material for: Pregnancy Outcomes in Women With Liver Cirrhosis: A National Prospective Cohort Study Using the UK Obstetric Surveillance System
Source: BJOG. 2025 Mar 13;132(7):935–43. doi: 10.1111/1471-0528.18107 (PMC12051225; doi:10.1111/1471-0528.18107)
Supplement: Supplementary file 3 — Figure S3. [file BJO-132-935-s001.docx]

Figure 3: Individual blood test values (median and interquartile ranges). Abbreviations: Plt – platelets; Hgb – haemoglobin; PT – prothrombin time; Alt – alanine transaminase The normal ranges for pregnancy are shaded in grey(21). The ‘worst’ result relates to the worst result during pregnancy.
