# Supplementary material for: Pregnancy Outcomes in Women With Liver Cirrhosis: A National Prospective Cohort Study Using the UK Obstetric Surveillance System
Source: BJOG. 2025 Mar 13;132(7):935–43. doi: 10.1111/1471-0528.18107 (PMC12051225; doi:10.1111/1471-0528.18107)
Supplement: Supplementary file 4 — Figure S4. [file BJO-132-935-s002.docx]

Supplementary Figure 4 a) AUROC curve: worst ALBI score in pregnancy predicts maternal decompensation as a composite of new ascites, encephalopathy and variceal bleed, AUROC 0.80 (p=0.03). 4b) AUROC curve: worst ALBI score in pregnancy predicts maternal ICU admission, AUROC, 0.82 (p=0.03). 4c) AUROC curve: worst ALBI score predicts pre-term birth, AUROC 0.74 (p=0.03).
